# Single dose of dexamethasone is not associated with postoperative recurrence and mortality in breast cancer patients: a propensity-matched cohort study

**DOI:** 10.1186/s12885-019-5451-5

**Published:** 2019-03-20

**Authors:** Myoung Hwa Kim, Dong Wook Kim, Seho Park, Joo Heung Kim, Ki Young Lee, Jisung Hwang, Young Chul Yoo

**Affiliations:** 1 Department of Anaesthesiology and Pain Medicine, Severance Hospital, Anaesthesia and Pain Research Institute, Yonsei University College of Medicine, 50-1 Yonsei-ro, Seodaemun-gu, Seoul, 03722 Korea; 2 Department of Policy Research Affairs, National Health Insurance Service Ilsan Hospital, Goyang, Gyeonggi-do Korea; 3 Division of Breast Surgery, Department of Surgery, Yonsei University College of Medicine, 50-1 Yonsei-ro, Seodaemun-gu, Seoul, 120-752 Republic of Korea; 4 Department of Surgery, National Health Insurance service Ilsan hospital, Goyang, Gyeonggi-do Korea

**Keywords:** Dexamethasone, Glucocorticoids, Breast cancer, Recurrence, Mortality, Immunosuppression

## Abstract

**Background:**

Dexamethasone is widely used in cancer patients despite the concern that perioperative glucocorticoids may potentially cause immunosuppression. However, studies on the influence of dexamethasone on cancer recurrence after curative surgery have produced conflicting results. The goal of our study was to compare postoperative recurrence-free survival and overall survival between patients with breast cancer who received perioperative dexamethasone and those who did not.

**Methods:**

The medical records of 2729 patients who underwent breast cancer surgery between November 2005 and December 2010 were reviewed. These patients were followed up until December 2015. The patients were categorised according whether they received a single dose of intravenous dexamethasone perioperatively or not. Cox regression analyses were conducted to evaluate any associations between dexamethasone usage with postoperative recurrence and mortality. Additionally, we performed a sensitivity test with propensity score matching to adjust for selection bias.

**Results:**

Among the 2628 patients, 236 (8.5%) received perioperative dexamethasone. No increasing risk for recurrence (hazard ratio [HR], 1.442; 95% confidence interval [CI], 0.969–2.145; *P* = 0.071) or mortality (HR, 1.256; 95% CI, 0.770–2.047; *P* = 0.361) after breast cancer surgery were identified in patients who received dexamethasone. Similarly, propensity score matching did not show significant associations in postoperative recurrence (HR, 1.389; 95% CI, 0.904–2.132; *P* = 0.133) or mortality (HR, 1.506; 95% CI, 0.886–2.561; *P* = 0.130) in patients who received dexamethasone.

**Conclusions:**

We found that a perioperative single dose of dexamethasone is not associated with increased recurrence or mortality after curative surgery in breast cancer patients.

## Background

Even though surgery is the best curative option for solid tumours, surgical manipulation still carries a risk of tumour spreading. Even after complete removal, tumour cells released during surgery may eventually lead to recurrence if they escape immune surveillance [1–3]. It is well known that corticosteroids induce generalized immunosuppression [4], and dexamethasone in particular can significantly suppress lymphokine production and cell proliferation [4], impair natural killer function [5, 6], and promote resistance to apoptosis in tumour cells [7].

However, many anaesthesiologists prefer to administer a single dose of dexamethasone because perioperative administration of this corticosteroid (4–10 mg intravenously) can reduce postoperative nausea/vomiting and surgical pain, and it can also enhance the quality of life during recovery, including the patient’s emotional state and physical independence [8, 9]. Despite its widespread use, there have been few clinical studies evaluating the influence of perioperative dexamethasone on oncological outcomes with conflicting results.

Breast cancer is one of the most common malignancies in women and is a leading cause of death globally. Although many studies on the treatment of breast cancer and prevention of postoperative recurrence have been performed, the 10-year survival rate in Western Europe is still 70% [10]. Therefore, it is necessary to investigate how dexamethasone, frequently used in the perioperative period, affects the outcomes of breast cancer, to address concerns about its immunosuppressive properties.

## Methods

### Subjects

Our protocol was approved by the Institutional Review Board and Hospital Research Ethics Committee of Severance Hospital, Yonsei University Health System (4-2017-0677). The board waived the requirement for informed consents because this study was retrospective. The data were collected from the electronic medical documents of patients who underwent curative surgery of breast cancer at a tertiary single institution between November 2005 and December 2010. All data were analysed except those of patients who underwent multiple surgeries simultaneously, lacked anaesthesia or surgical information, or received steroid therapy for any reason.

### Data collection

Retrieved demographic data of the patients included age, sex, body mass index (BMI), co-morbidities, induction agents, anaesthetic agents, and antiemetic agents and analgesics. Dexamethasone was administered perioperatively at the discretion of the anaesthesiologists. We also collected surgical information including the surgical procedure and duration, expressed cellular receptors, tumour-node-metastasis (TNM) staging, tumour type, histological type, and any administered chemotherapy or radiotherapy. Clinicopathological parameters, including levels of oestrogen receptor (ER), progesterone receptor (PR), and human epidermal growth factor receptor (HER) 2, were obtained from the registry database. Tumours with ≥1% of their cells exhibiting ER and/or PR staining were considered positive for these receptors. In addition, we used the American Joint Committee on Cancer 7th edition criteria to identify the TNM staging [11]. Local (or regional) metastasis was determined as tumour recurrence in the ipsilateral breast, regional lymph nodes, and/or chest wall. Every 6–12 months, the patients were observed clinically, which included taking a medical history, performing a physical examination, and performing laboratory and imaging tests to detect any cancer relapse. Any recurrence at a distant site, including the contralateral axillary or supraclavicular lymph nodes, was defined as a distant metastasis. Recurrence-free survival was calculated from the date of surgery to the date on which loco-regional or distant metastasis was first detected. The postoperative overall survival was assessed from the first date of curative surgery to the last follow-up date or death from any cause.

### Statistical analyses

Patients who received dexamethasone and those who did not were compared using Student’s t-test for continuous variables and the *X*^2^ test for categorical variables. *P*-values less than 0.05 were considered statistically significant. All potential confounders associated with recurrence and mortality after breast cancer surgery, which were chosen based on their clinical significance as reported in the literature were analysed using competing risk and Cox regression analyses, respectively. First, we performed univariate analysis to identify potential risk factors for postoperative recurrence and mortality; those with *P*-values of < 0.1 were subjected to multivariate analysis, following which hazard ratios (HRs) and the associated 95% confidence intervals (CIs) were estimated. Fine and Gray competing risk analysis was performed for the recurrence with death as a competing risk. The univariate associations between dexamethasone usage and overall survival were assessed using Kaplan-Meier survival analysis together with the log-rank test. We also conducted a sensitivity test to assess the robustness of our findings with respect to the relationship between dexamethasone and each of recurrence and mortality, wherein we performed propensity score matching. Variables were adjusted to factors including BMI, hypertension, and diabetes mellitus, and the score was calculated with logistic regression. A greedy heuristic algorithm was used to identify the optimally matched groups without drop-outs; this excluded cases with differences exceeding twice the standard deviation (SD) during matching similar propensity scores. As a result, 1-to-5 matching was chosen because it carried the strongest statistical power. All statistical analyses were conducted with SAS version 9.4 (SAS Institute Inc., Cary, NC, USA) except for Kaplan-Meier curves, which were constructed using the R package version 3.0.2 (http://www.r-project.org).

## Results

### Subjects

We collected the data from 2729 patients who underwent surgery following their diagnosis with breast cancer during the study period. Among them, 63 patients who underwent multiple surgeries, 21 with unclear anaesthetic methods, and 17 who received preoperative steroid therapy were excluded. Ultimately, 2628 patients were analysed in our study. The patients were followed up to December 2015. The mean (SD) follow-up period for our study population was 70.1 (23.1) months.

### Patients’ demographic data

A comparison of the characteristics of patients who received dexamethasone (*N* = 236) and those who did not (*N* = 2392) is shown in Table 1. These two groups had similar baseline demographic data. However, there were significant differences with respect to anaesthetic agent, induction agent, nitric oxide use, antiemetic use, surgical procedure, and chemotherapy/radiotherapy administration between the two groups.

**Table 1:** Comparison of characteristics between patients who received dexamethasone and those who did not

	Non-dexamethasone (*N* = 2392)	Dexamethasone (*N* = 236)	*P*-value
Demographic data			
Age (years)	50.1 ± 10.3	49.5 ± 9.3	0.418
BMI (kg m^−2^)	23.3 ± 3.1	23.4 ± 3.1	0.764
Comorbidity			
HTN	473 (19.8)	44 (18.6)	0.732
DM	172 (7.2)	14 (5.9)	0.594
Cardiac disease	60 (2.5)	4 (1.7)	0.656
Pulmonary disease	46 (1.9)	6 (2.5)	0.463
Endocrine disease	109 (4.6)	14 (5.9)	0.332
Renal disease	16 (0.7)	1 (0.4)	> 0.999
Liver disease	16 (0.7)	2 (0.8)	0.673
Neurological disease	40 (1.7)	4 (1.7)	> 0.999
Others	14 (0.6)	3 (1.3)	0.191
Anaesthetic factors			
Anaesthetic agent			0.001
Volatiles			
Sevoflurane	1461 (61.1)	141 (59.7)	
Desflurane	613 (25.6)	47 (19.9)	
Isoflurane	242 (10.1)	27 (11.4)	
Enflurane	31 (1.3)	10 (4.2)	
TIVA	45 (1.9)	11 (4.7)	
Induction agents			0.029
Propofol	1856 (77.6)	168 (71.2)	
Barbiturate	536 (22.4)	68 (28.8)	
N_2_O	176 (7.4)	28 (11.9)	0.021
Premedication^a^	1585 (66.3)	169 (71.6)	0.111
Antiemetic	2069 (86.5)	180 (76.3)	< 0.001
Rescue analgesics	2297 (96.0)	225 (95.3)	0.602
Hypertensive events	145 (6.1)	15 (6.4)	0.886
Hypotensive events	343 (14.3)	25 (10.6)	0.140
Colloid administration	36 (1.5)	6 (2.5)	0.267
RBC transfusion	12 (0.5)	0 (0)	0.616
Surgical factors			
Surgical procedure			0.014
BCS	1156 (48.3)	94 (39.8)	
Mastectomy	1218 (51.7)	170 (60.2)	
Surgical duration (min)	207.2 ± 131.0	212.5 ± 108.5	0.487
TNM staging			0.974
1	1126 (47.1)	113 (47.9)	
2	876 (36.7)	86 (36.4)	
3	387 (16.2)	37 (15.7)	
Receptors			
Oestrogen	1652 (69.1)	173 (73.3)	0.183
Progesterone	1506 (63.0)	151 (64.0)	0.778
HER2	658 (27.5)	60 (25.4)	0.540
Histological analysis			0.118
Well-differentiated	508 (21.2)	48 (20.3)	
Moderately differentiated	1058 (44.2)	105 (44.5)	
Poorly differentiated	584 (24.4)	48 (20.3)	
Others	242 (10.1)	35 (14.8)	
Tumour types			0.818
IDC	2105 (88.0)	212 (87.7)	
ILC	85 (3.6)	10 (4.2)	
Others	202 (8.4)	19 (8.1)	
Chemotherapy	1619 (67.7)	179 (75.8)	0.010
Radiotherapy	1559 (65.2)	137 (58.1)	0.032

Data are presented as mean ± standard deviation, or number (percentage)

*BMI* body mass index, *HTN* hypertension, *DM* diabetes mellitus, *TIVA* total intravenous anaesthesia, *N*_*2*_*O* nitrous oxide, *RBC* red blood cell, *BCS* breast conserving surgery, *TNM* tumour–node–metastasis, *HER2* human epidermal growth factor receptor 2, *IDC* invasive ductal carcinoma, *ILC* invasive lobular carcinoma

^a^Premedication: Midazolam 0.03 mg kg^−1^ was administered

### Risk factors for postoperative recurrence in breast cancer surgery

**Fig. 1:**
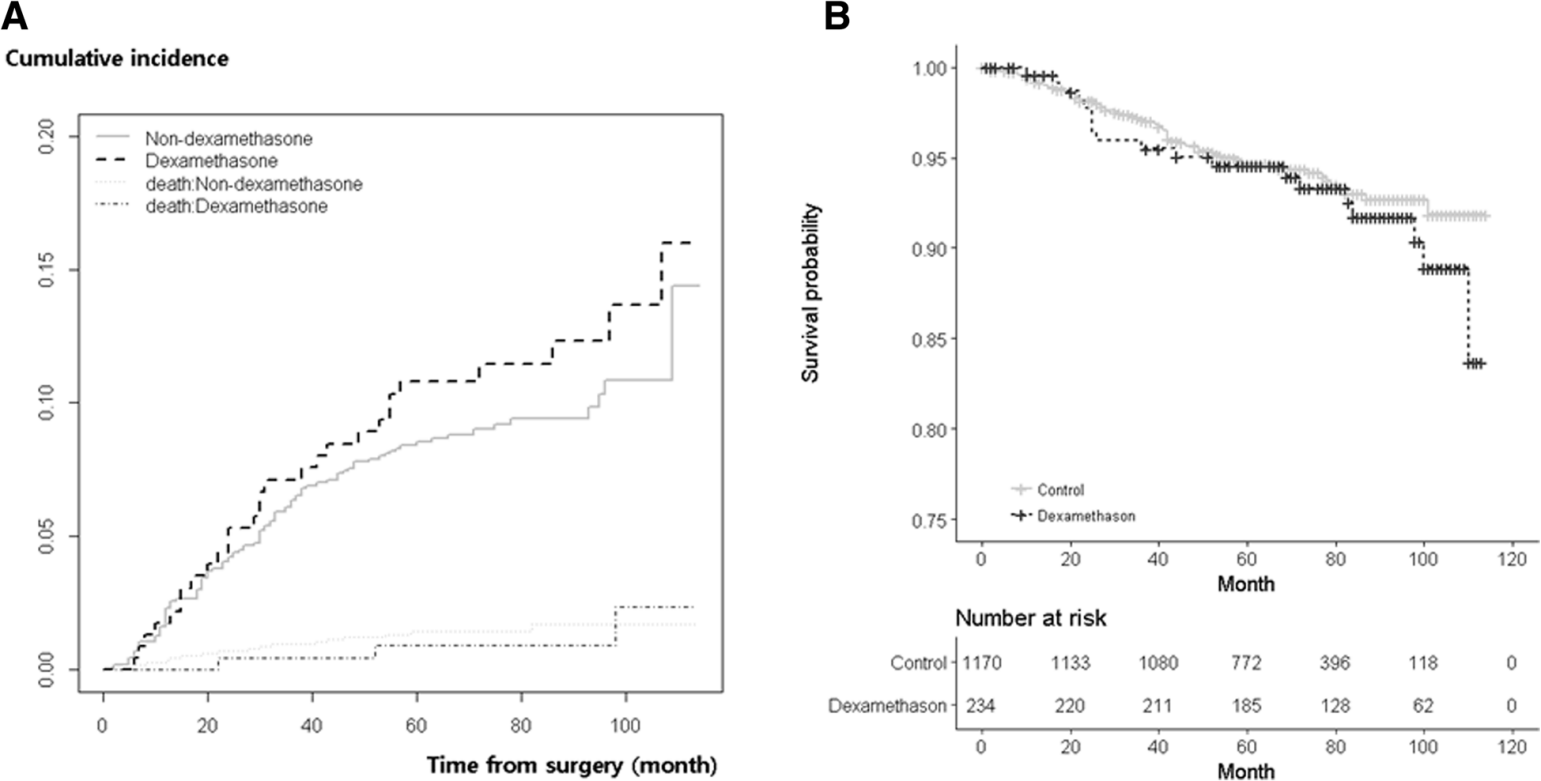
**a** Probability of recurrence-free survival following breast cancer surgery as a function of dexamethasone administration. **b** Probability of postoperative overall survival in breast cancer patients as a function of dexamethasone administration

Table 2 presents the factors that affected postoperative breast cancer recurrence as revealed by competing risk analyses. Multivariate analyses revealed that cancer recurrence was not associated with perioperative dexamethasone administration (hazard ratio [HR], 1.442; 95% confidence interval [CI], 0.969–2.145; *P* = 0.071). Table 3 details the propensity 1-to-5 matching analysis for our primary findings. This also showed no association between dexamethasone and postoperative recurrence (HR, 1.389; 95% CI, 0.904–2.132; *P* = 0.133). Other anaesthetic factors also were not correlated with the recurrence. The type of surgical procedure and higher TNM staging (stage 2 and 3) were significantly associated with breast cancer recurrence. Figure 1a shows the recurrence-free survival probabilities following breast cancer surgery according to dexamethasone administration; this showed no significant influence due to dexamethasone (*P* = 0.295).

**Table 2:** Competing risk analyses of factors associated with cancer recurrence after surgery for breast cancer

Variables	Univariate				Multivariate			
	HR	95% CI		*P*-value	HR	95% CI		*P*-value
Dexamethasone								
No	1 (ref)				1 (ref)			
Yes	1.348	0.912	1.994	0.134	1.442	0.969	2.145	0.071
Age	0.989	0.976	1.002	0.089				
Age (years)								
< 40	1 (ref)				1 (ref)			
40–49	0.673	0.469	0.967	0.032	0.866	0.598	1.254	0.447
50–59	0.705	0.482	1.032	0.072	0.779	0.526	1.154	0.213
60–69	0.665	0.421	1.05	0.080	0.703	0.416	1.188	0.188
≥70	0.563	0.254	1.249	0.158	0.591	0.247	1.414	0.237
BMI	0.99	0.948	1.034	0.654				
BMI (kg m^−2^)								
< 18.4	1.033	0.523	2.039	0.927	0.835	0.421	1.658	0.607
18.4–22.9	1 (ref)				1 (ref)			
23–24.9	1.026	0.739	1.423	0.880	1.100	0.791	1.530	0.572
25–29.9	1.033	0.749	1.426	0.842	1.021	0.738	1.411	0.901
≥30	0.73	0.298	1.789	0.491	0.626	0.254	1.546	0.310
HTN								
No	1 (ref)				1 (ref)			
Yes	0.952	0.686	1.322	0.770	1.045	0.714	1.531	0.820
DM								
No	1 (ref)				1 (ref)			
Yes	1.132	0.700	1.832	0.612	1.158	0.681	1.968	0.588
Anaesthetic agents								
TIVA	1 (ref)				1 (ref)			
Volatile	1.247	0.550	2.830	0.597	1.072	0.470	2.441	0.869
N_2_O								
No	1 (ref)							
Yes	1.325	0.879	1.997	0.179				
Transfusion								
No	1 (ref)							
Yes	2.591	0.644	10.426	0.180				
Premedication^a^								
No	1 (ref)							
Yes	0.878	0.670	1.150	0.345				
Surgical procedure								
BCS	1 (ref)				1 (ref)			
Mastectomy	2.516	1.883	3.360	< 0.001	1.934	1.429	2.618	< 0.001
TNM staging								
1	1 (ref)				1 (ref)			
2	2.397	1.674	3.431	< 0.001	1.778	1.168	2.706	0.007
3	7.395	5.217	10.48	< 0.001	5.300	3.433	8.183	< 0.001
Oestrogen receptor								
No	1 (ref)				1 (ref)			
Yes	0.576	0.443	0.748	< 0.001	0.886	0.597	1.315	0.549
Progesterone receptor								
No	1 (ref)				1 (ref)			
Yes	0.605	0.467	0.783	< 0.001	0.835	0.571	1.222	0.355
Chemotherapy								
No	1 (ref)				1 (ref)			
Yes	3.265	2.213	4.818	< 0.001	0.958	0.580	1.582	0.866
Radiotherapy								
No	1 (ref)							
Yes	1.211	0.917	1.597	0.177				

*CI* confidence interval, *HR* hazard ratio, *BMI* body mass index, *HTN* hypertension, *DM* diabetes mellitus, *TIVA* total intravenous anaesthesia, *N*_*2*_*O* nitrous oxide, *BCS* breast conserving surgery, *TNM* tumour-node-metastasis

^a^Premedication: Midazolam 0.03 mg kg^−1^ was administered

**Table 3:** Factors associated with postoperative cancer recurrence in patients with breast cancer after propensity score matching

Variables	Univariate				Multivariate			
	HR	95% CI		*P*-value	HR	95% CI		*P*-value
Dexamethasone								
No	1 (ref)				1 (ref)			
Yes	1.251	0.822	1.903	0.295	1.389	0.904	2.132	0.133
Age	0.982	0.964	1.000	0.055				
Age (years)								
< 40	1 (ref)				1 (ref)			
40–49	0.736	0.466	1.162	0.188	1.053	0.655	1.690	0.832
50–59	0.572	0.347	0.944	0.029	0.614	0.364	1.037	0.068
60–69	0.685	0.361	1.301	0.248	0.603	0.289	1.258	0.177
≥70	0.613	0.186	2.017	0.421	0.453	0.126	1.628	0.225
BMI	0.977	0.922	1.035	0.436				
BMI (kg m^−2^)								
< 18.4	1.349	0.542	3.358	0.520	1.477	0.585	3.730	0.409
18.4–22.9	1 (ref)				1 (ref)			
23–24.9	1.159	0.765	1.757	0.485	1.321	0.866	2.015	0.196
25–29.9	0.919	0.597	1.417	0.703	1.032	0.667	1.597	0.888
≥30	0.326	0.045	2.353	0.266	0.345	0.047	2.524	0.295
HTN								
No	1 (ref)				1 (ref)			
Yes	1.109	0.718	1.713	0.642	1.270	0.761	2.118	0.360
DM								
No	1 (ref)				1 (ref)			
Yes	1.438	0.755	2.740	0.269	1.502	0.742	3.040	0.259
Anaesthetic agents								
TIVA	1 (ref)				1 (ref)			
Volatile	0.575	0.235	1.406	0.225	0.782	0.312	1.960	0.600
N_2_O								
No	1 (ref)							
Yes	1.515	0.919	2.497	0.103				
Transfusion								
No	1 (ref)							
Yes	1.894	0.265	13.547	0.525				
Premedication^a^								
No	1 (ref)							
Yes	0.873	0.611	1.249	0.458				
Surgical procedure								
BCS	1 (ref)				1 (ref)			
Mastectomy	2.119	1.458	3.078	< 0.001	1.659	1.121	2.457	0.012
TNM staging								
1	1 (ref)				1 (ref)			
2	2.454	1.503	4.006	< 0.001	2.160	1.223	3.814	0.008
3	8.215	5.121	13.177	< 0.001	7.780	4.303	14.064	< 0.001
Oestrogen receptor								
No	1 (ref)				1 (ref)			
Yes	0.564	0.400	0.796	0.001	0.783	0.463	1.325	0.363
Progesterone receptor								
No	1 (ref)				1 (ref)			
Yes	0.572	0.407	0.803	0.001	0.818	0.491	1.363	0.441
Chemotherapy								
No	1 (ref)				1 (ref)			
Yes	1.870	1.293	2.706	0.001	0.669	0.350	1.279	0.224
Radiotherapy								
No	1 (ref)							
Yes	1.683	1.139	2.486	0.009				

*CI* confidence interval, *HR* hazard ratio, *BMI* body mass index, *HTN* hypertension, *DM* diabetes mellitus, *TIVA* total intravenous anaesthesia, *N*_*2*_*O* nitrous oxide, *BCS* breast conserving surgery, *TNM* tumour-node-metastasis

In 1 to 5 propensity matching process, the basic characteristics of 1224 subjects in the control group were considered to be different from the dexamethasone group and excluded from the final matching analyses. Consequently, 234 patients who received dexamethasone and 1170 patients who did not receive dexamethasone were included

^a^Premedication: Midazolam 0.03 mg kg^−1^ was administered

### Related-factors for postoperative mortality in breast cancer surgery

Table 4 presents the factors that potentially influence postoperative mortality in patients with breast cancer following Cox regression. Multivariate analyses revealed no association between perioperative dexamethasone administration and mortality (HR, 1.389; 95% CI, 0.904–2.132; *P* = 0.133). Instead, this was significantly associated with old age (≥70 years), transfusion, the type of surgical procedure, higher TNM staging (stage 3), and progesterone receptor-positive status. Table 5 details the association between dexamethasone and postoperative mortality as identified after the propensity 1-to-5 matching. While there was no association between dexamethasone and postoperative mortality (HR, 1.506; 95% CI, 0.886–2.561; *P* = 0.130), the type of surgical procedure, higher TNM stage (≥3), and progesterone receptor were significantly associated with postoperative mortality. Figure 1b shows the postoperative overall survival probability in patients according to dexamethasone use; this showed that dexamethasone did not significantly influence the overall survival (*P* = 0.442).

**Table 4:** Univariate and multivariate Cox regression analyses of factors associated with mortality after surgery for breast cancer

Variables	Univariate				Multivariate			
	HR	95% CI		*P*-value	HR	95% CI		*P*-value
Dexamethasone								
No	1 (ref)				1 (ref)			
Yes	1.051	0.652	1.696	0.837	1.256	0.770	2.047	0.361
Age	1.028	1.013	1.042	< 0.001				
Age (years)								
< 40	1 (ref)				1 (ref)			
40–49	0.586	0.369	0.931	0.024	0.772	0.480	1.240	0.285
50–59	0.852	0.539	1.346	0.493	0.922	0.575	1.479	0.736
60–69	1.175	0.716	1.929	0.523	1.151	0.654	2.027	0.625
≥70	2.713	1.534	4.798	< 0.001	2.427	1.220	4.830	0.012
BMI	0.993	0.947	1.041	0.758				
BMI (kg m^−2^)								
< 18.4	1.564	0.810	3.021	0.183	1.271	0.653	2.474	0.480
18.4–22.9	1 (ref)				1 (ref)			
23–24.9	1.14	0.788	1.649	0.488	1.180	0.813	1.715	0.384
25–29.9	1.016	0.696	1.483	0.934	0.963	0.657	1.413	0.847
≥30	1.209	0.527	2.775	0.654	1.265	0.545	2.939	0.584
HTN								
No	1 (ref)				1 (ref)			
Yes	1.564	1.128	2.166	0.007	1.048	0.703	1.563	0.817
DM								
No	1 (ref)				1 (ref)			
Yes	1.810	1.148	2.852	0.0107	1.217	0.732	2.023	0.450
Anaesthetic agents								
TIVA	1 (ref)				1 (ref)			
Volatile	0.497	0.123	2.017	0.328	3.036	0.738	12.485	0.124
N_2_O								
No	1 (ref)							
Yes	1.388	0.892	2.158	0.146				
Transfusion								
No	1 (ref)				1 (ref)			
Yes	7.527	2.792	20.296	< 0.001	5.551	1.980	15.559	0.001
Premedication^a^								
No	1 (ref)							
Yes	0.817	0.602	1.108	0.193				
Surgical procedure								
BCS	1 (ref)				1 (ref)			
Mastectomy	3.073	2.169	4.355	< 0.001	2.244	1.556	3.236	< 0.001
TNM staging								
1								
2	1.902	1.275	2.838	0.002	1.591	0.994	2.545	0.053
3	6.312	4.318	9.226	< 0.001	5.435	3.338	8.850	< 0.001
Oestrogen receptor								
No	1 (ref)				1 (ref)			
Yes	0.398	0.297	0.533	< 0.001	0.758	0.488	1.177	0.217
Progesterone receptor								
No	1 (ref)				1 (ref)			
Yes	0.369	0.274	0.496	< 0.001	0.565	0.367	0.870	0.010
Chemotherapy								
No	1 (ref)				1 (ref)			
Yes	1.870	1.293	2.706	< 0.001	0.674	0.403	1.128	0.133
Radiotherapy								
No	1 (ref)							
Yes	0.922	0.682	1.246	0.597				

*CI* confidence interval, *HR* hazard ratio, *BMI* body mass index, *HTN* hypertension, *DM* diabetes mellitus, *TIVA* total intravenous anaesthesia, *N*_*2*_*O* nitrous oxide, *BCS* breast conserving surgery, *TNM* tumour-node-metastasis

^a^Premedication: Midazolam 0.03 mg kg^−1^ was administered

**Table 5:** Factors associated with postoperative mortality in patients with breast cancer after propensity score matching

Variables	Univariate				Multivariate			
	HR	95% CI		*P*-value	HR	95% CI		*P*-value
Dexamethasone								
No	1 (ref)				1 (ref)			
Yes	1.223	0.733	2.041	0.442	1.506	0.886	2.561	0.130
Age	1.005	0.983	1.028	0.636				
Age (years)								
< 40	1 (ref)				1 (ref)			
40–49	0.607	0.331	1.113	0.107	0.979	0.517	1.855	0.949
50–59	0.763	0.416	1.400	0.382	0.778	0.411	1.473	0.440
60–69	1.165	0.574	2.364	0.673	0.962	0.419	2.208	0.927
≥70	1.087	0.318	3.714	0.894	0.866	0.226	3.323	0.834
BMI	0.991	0.925	1.063	0.810				
BMI (kg m^−2^)								
< 18.4	0.852	0.542	3.358	0.520	0.759	0.179	3.216	0.709
18.4–22.9	1 (ref)				1 (ref)			
23–24.9	1.449	0.765	1.757	0.485	1.647	0.994	2.730	0.053
25–29.9	1.012	0.597	1.417	0.703	1.008	0.584	1.740	0.976
≥30								
HTN								
No	1 (ref)							
Yes	1.692	1.052	2.723	0.030				
DM								
No	1 (ref)							
Yes	1.775	0.858	3.672	0.122				
Anaesthetic agents								
TIVA	1 (ref)				1 (ref)			
Volatile	1.050	0.258	4.272	0.946	1.400	0.332	5.892	0.647
N_2_O								
No	1 (ref)							
Yes	1.523	0.840	2.763	0.166				
Transfusion								
No	1 (ref)				1 (ref)			
Yes	6.113	1.503	24.857	0.011	3.063	0.6900	13.600	0.141
Premedication^a^								
No	1 (ref)							
Yes	0.796	0.515	1.231	0.305				
Surgical procedure								
BCS	1 (ref)				1 (ref)			
Mastectomy	2.699	1.652	4.408	< 0.001	2.289	1.367	3.833	0.002
TNM staging								
1	1 (ref)				1 (ref)			
2	2.403	1.293	4.466	0.006	1.553	0.774	3.117	0.216
3	8.587	4.770	15.458	< 0.001	5.509	2.732	11.106	< 0.001
Oestrogen receptor								
No	1 (ref)				1 (ref)			
Yes	0.338	0.222	0.514	< 0.001	0.699	0.374	1.305	0.261
Progesterone receptor								
No	1 (ref)				1 (ref)			
Yes	0.280	0.180	0.435	< 0.001	0.428	0.228	0.803	0.008
Chemotherapy								
No	1 (ref)				1 (ref)			
Yes	4.667	2.156	10.103	< 0.001	1.381	0.549	3.474	0.493
Radiotherapy								
No	1 (ref)							
Yes	1.473	0.926	2.343	0.102				

*CI* confidence interval, *HR* hazard ratio, *BMI* body mass index, *HTN* hypertension, *DM* diabetes mellitus, *TIVA* total intravenous anaesthesia, *N*_*2*_*O* nitrous oxide, *BCS* breast conserving surgery, *TNM* tumour-node-metastasis

In 1 to 5 propensity matching process, the basic characteristics of 1224 subjects in the control group were considered to be different from the dexamethasone group and excluded from the final matching analyses. Consequently, 234 patients who received dexamethasone and 1170 patients who did not receive dexamethasone were included

^a^Premedication: Midazolam 0.03 mg kg^−1^ was administered

### Sub-group analyses for association between dexamethasone and subtypes of breast cancer

In the sub-group analyses to specify association between dexamethasone and subtypes of breast cancer including ER-dependency, PR-dependency or TNM staging, there was no association between dexamethasone and the recurrence or mortality in ER-dependent breast cancer. Instead, dexamethasone was significantly associated with the risk of mortality in PR-positive breast cancer (HR 2.637, 95% CI 1.106–6.288, *p* = 0.029). Moreover, dexamethasone showed a significant tendency to increase risk of the recurrence in TNM stage 1of breast cancer (HR 2.976, 95% CI 1.106–6.288, *p* = 0.002).

## Discussion

Dexamethasone is an effective and widely used agent that is administered perioperatively to improve the quality of recovery after surgery. However, dexamethasone can also induce immunosuppression and may cause tumour cells to escape from the immune system. As a patient’s perioperative management is only handled by the anaesthesiologist, the choice of anaesthetic agent may critically affect post-surgical outcomes. Hence, anaesthesiologists should consider the possibility of immunosuppression before administering dexamethasone. From this perspective, investigating the effects of perioperative dexamethasone administration on postoperative oncologic outcomes, in patients who were already immunocompromised due to the perioperative environment, is very important. In our study, we found that perioperative dexamethasone was not associated with recurrence or mortality of patients after breast cancer surgery.

Stress response following surgery is part of the systemic reaction to an injury, and it encompasses a wide range of immunological, endocrinological, and haematological effects [12]. Importantly, the inevitable immunosuppression during the perioperative period can allow tumour cells to evade the immune system, leading to recurrence in patients who are prone to undetectable micrometastases [13], even though surgical resection of a tumour remains the optimal curative treatment. Previous investigations suggest that perioperative impairment of the immune system increases the risk of cancer recurrence in patients undergoing oncologic surgery [14, 15]. Glucocorticoids are involved in postoperative suppression of natural killer cell activity [16]; their role in immunosuppression includes the significant suppression of lymphokine production and cell proliferation [4], impairment of natural killer function [6], and rendering tumour cells more resistant to apoptosis [7]. Glucocorticoids are also known to induce proliferation in normal cells such as erythroid progenitor cells and fibroblasts [17, 18]; and previous studies have shown that low concentrations of dexamethasone also can induce in vitro proliferation of cancer cells such as those derived from glioma [19], astrocytoma [20], and Kaposi’s sarcoma [21]. Hence, even though dexamethasone-induced immunosuppression may be temporary, it can aggravate tumour cell evasion of immune surveillance.

The issue of perioperative dexamethasone-induced immunosuppression influencing oncological outcomes has recently been investigated by other groups. Yu and colleagues retrospectively studied 515 patients who underwent rectal cancer surgery and found a higher rate of cancer recurrence in patients who received dexamethasone [22]. Moreover, patients receiving a single dose of dexamethasone before undergoing colon cancer surgery were shown to have a potentially increased risk of distant recurrence [23]. On the other hand, a retrospective study of 309 women who underwent endometrial cancer surgery showed that dexamethasone that was administered to prevent postoperative nausea and vomiting was not associated with an increased risk of cancer recurrence or with altered progression-free or overall survival compared to patients who did not receive dexamethasone [24]. Additionally, De Oliveira et al. [25] demonstrated that perioperative dexamethasone had no effect on recurrence in patients diagnosed with ovarian cancer, although they did not report survival data. Another study found that perioperative dexamethasone might improve postoperative survival in human pancreatic adenocarcinoma [26].

Our finding that perioperative dexamethasone appears to have no significant effect on postoperative outcomes including recurrence free and overall survival of patients who underwent breast cancer surgery may be explained as follows: Dexamethasone has been shown to reduce the perioperative stress response [27, 28]; therefore, the direct immunosuppression caused by dexamethasone may be counteracted by its protective effect on the normal stress response to surgical stimuli. Dexamethasone also has pro-apoptotic properties that may lengthen the survival of patients with certain types of cancer [29, 30]. Glucocorticoids can destroy cancerous lymphoid cells, and are thus essential for treating lymph node tissue malignancies.

In contrast to these favourable characteristics of dexamethasone, glucocorticoids in general may also induce the growth of malignant solid tumours and increase their dissemination as a consequence of decreasing inter-cell adhesiveness and enhancing tissue permeability. In this regard, two recent reports were published on the relationship between glucocorticoid use and breast cancer risk. They described no evidence of glucocorticoid use influencing breast cancer recurrence in Danish populations [31, 32]. However, these studies included breast cancer patients regardless of whether they underwent surgery, and also investigated various types of glucocorticoids combined. To the best of our knowledge, ours is the first study to investigate the impact of perioperative dexamethasone on postoperative recurrence and mortality in patients with breast cancer, and to determine whether single-dose dexamethasone is safe in patients undergoing breast cancer surgery.

Based on our findings, chemotherapy was not significantly associated with postoperative recurrence and mortality in the multivariate analyses, in contrast to univariate analysis results. This may be because of confounding effects associated with chemotherapy and TNM staging, as chemotherapy is administered according to the TNM stage in breast cancer patients. However, we could not avoid investigating chemotherapy and TNM stage together in our multivariate analysis model, as both were critical for determining the effect of dexamethasone on postoperative outcomes. Moreover, a higher TNM stage remained a critical risk factor for postoperative recurrence and mortality despite chemotherapy. Although the administration of dexamethasone to subjects with chemotherapy may lead to worsened prognoses in patients with breast cancer, it likely has little impact on the perioperative immunity of patients because surgery is usually scheduled 1 month after completing chemotherapy at our institution. Moreover, while the antiemetic regimen is prescribed according to the degree of nausea experienced by patients, dexamethasone is not a routinely administered agent. Furthermore, a large cohort study of breast cancer patients [32] showed no evidence of an association between preoperative systemic, inhaled, or intestinal-acting glucocorticoids and breast cancer recurrence. As our study was designed to investigate the effect of a single dose of dexamethasone during anaesthesia on oncologic outcomes, we did not include the data of dexamethasone administered for neoadjuvant chemotherapy. Additionally, we performed sub-group analyses to find out more association between dexamethasone and subtypes of breast cancer such as ER-dependency, PR-dependency, or TNM staging. In this result, we found that dexamethasone is associated with the risk of mortality in PR-dependent breast cancer and recurrence in TNM stage 1 after breast cancer surgery. This detrimental effect of dexamethasone in TNM stage 1 and PR-dependent breast cancer requires further studies with sufficient sample size.

The present study is limited by its retrospective nature. Our patients were not randomised, and the clinical protocol was not standardised; therefore, selection bias and the effects of unmeasured confounding variables cannot be excluded. Moreover, the factors that influenced care providers to administer or forgo perioperative dexamethasone are unknown, although such decisions were unlikely to be associated with tumour status. Even though single-dose perioperative dexamethasone administration has known to have no association with an increased risk of acute postoperative complications including wound infection, the lack of information regarding dexamethasone-related adverse effects other than recurrence or mortality was considered a weak point of the study. There may be limited strength to the conclusion because of the relatively small population who received dexamethasone compared with the large total sample size. Nevertheless, the reliability of our data was buttressed by the retrospective design to some extent, because our homogeneous patients comprising 2628 women were managed perioperatively in a similar manner at the same hospital. Above all, our analyses may have the statistical power by the sensitivity test of 1-to-5 propensity score matching, which led to well-matched groups and minimized the potential for significant confounding. Although randomised clinical trials are required for definitive conclusions, our results from post-propensity score matching ought to add valuable insight despite the study’s limitations.

## Conclusions

In conclusion, our results suggest that a single dose of perioperative dexamethasone does not increase the risk of recurrence in patients after breast cancer surgery, and it appears to be safe in this patient population.

## Abbreviations

BMI: Body mass index
CI: Confidence interval
ER: Oestrogen receptor
HER: Human epidermal growth factor receptor 2
HR: Hazard ratio
PR: Progesterone receptor
SD: Standard deviation
TNM: Tumour-node-metastasis
